# Editorial: Conscious perception of nature and times of silence as resources to improve public mental health

**DOI:** 10.3389/fpubh.2024.1397593

**Published:** 2024-03-21

**Authors:** Arndt Büssing, Klaus Baumann

**Affiliations:** ^1^Institut für Integrative Medizin, Universität Witten/Herdecke, Herdecke, Germany; ^2^Theologische Fakultät, Albert Ludwigs Universität Freiburg, Freiburg im Breisgau, Germany

**Keywords:** nature, times of quietness, restoration, mental health, experience

In recent years, nature as a resource has become a topic of interest not only in health psychology research. Stressed people, especially in urban environments, seek for timeouts to relax. They need places where the soul can “recharge”, and the body can rest. These places are for most people green and blue spaces in a beautiful environment (“nature”) (1). However, others want to experience and expose themselves in the great outdoors (“wilderness”) as a contrast to their routines of everyday activities on their job.

It is not surprising that the Romantic era (at the end of the 18th and start of the 19th century)—as a counter-movement to enlightenment by reason and to the starting period of industrialization with its social insecurities and individual difficulties in finding meaning and orientation in life—was associated with a longing for the immediate and mysterious in untouched nature and vastness. This idealized Nature could be considered as a concrete or idealized place to escape from the dreariness of everyday life concerns.

The Frontiers in Public Health's Research Topic “*Conscious Perception of Nature and Times of Silence as Resources to Improve Public Mental Health*” addresses these issues in different ways.

The article “*Experience of nature and times of silence as a resource to cope with the COVID-19 pandemic and their effects on psychological wellbeing—Findings from a continuous cross-sectional survey in Germany*” by Büssing et al. found that being in nature and enjoying silence, and related feelings of awe and gratitude were relevant resources during the COVID-19 pandemic. Perception of nature could be regarded as a sensitizer of positive experiences, particularly during difficult phases of life; and these could be trained to stabilize wellbeing and thus contribute to public health.

The article “*Positive Religious Coping Acts Through Perception of Nature and Silence in its Association with Well-Being and Life Satisfaction Among Polish Catholics*” by Skalski-Bednarz et al. addressed the associations between religious coping, perceptions of nature and silence, and quality of life. The group found that perceptions of nature and reflective times of silence “partially mediated the associations of positive religious coping with wellbeing and life satisfaction”. Based on their findings they suggest the need for “interventions that help people develop an ability or awareness for nature as an exceptional encounter”. Particularly people with a religious/spiritual awareness may benefit from such an awareness of nature and silence.

The article “*The impact of gratitude on connection with nature: the mediating role of positive emotions of self-transcendence*” by Chen et al. addresses being connected with nature and related attitudes and perceptions. The authors found that emotions of self-transcendence partially mediated the link between gratitude and connection to nature, and further that “self-transcendence and connection to nature were fully and continuously mediated”. They underline that gratitude may have an indirect effect on both “willingness to participate in environmental protection” and further “willingness to sacrifice for the environment”. This means that being grateful may transcend the own ego, and facilitates a connectedness to nature and a motivation to care for and protect the environment.

The group Glavas et al. reported in their article “*Inner Peace needs of male psychiatric patients in post-war Croatia are associated with their needs to clarify open issues in their life and their needs for forgiveness*” that inner peace needs scored highest in male patients treated with PTSD diagnoses as compared to men with other psychiatric diagnoses (3). Their findings indicate patients' intention to let go of their disturbing experiences of the Balkan war and to find “*states of inner peace, particularly at specific places of quietness and peace*”. Apart from psychotherapeutic treatment, this would indicate that further support options like (e.g.) spiritual care interventions are needed for this group of post-war veterans.

The article “*Effects of immersion in a simulated natural environment on stress reduction and emotional arousal: A systematic review and meta-analysis*” by Li et al. asks whether and to what extend audio-visual exposure to simulated nature influences mental health. Their systematic review confirms that simulating nature experiences is related to positive affect, vigor, and calmness on the one hand, and to lower stress, mood disturbance, tension, fatigue, anxiety, depression, confusion, and anger on the other side. This is particularly important to support people with limited access to nature exposure or physical impairment.

In addition to the established therapeutic potential of gardening, the qualitative study “*Traces of health – A landscape design task as a diagnostic aid for detecting mental burden? A qualitative focus group study*” by Niedermann et al. revealed that gardening and landscape design may contain diagnostic elements. Their preliminary findings indicate the association of movement and design patterns with mental burden and justify further research on the relevance of landscape design tasks for people with mental burden.

All these findings add important aspects to the broad topic of the relationship between experiences of nature and mental health, which stimulates further research. Particularly the experiential aspects of nature exposure are important: What do people experience at different places and in different situations, and how does this impact their feelings, attitudes and behaviors? How can the positive effects of nature experience on mental health issues be used to promote public health?—These are only very few of many more questions worth addressing in future investigations, and thus we are sure that further exciting research can be expected that helps to sharpen awareness that we only have one world in which to live our lives as one human family.

## Author contributions

AB: Writing – original draft. KB: Writing – review & editing.
